# A *SEPSECS* mutation in a 23-year-old woman with microcephaly and progressive cerebellar ataxia

**DOI:** 10.1007/s10545-018-0151-x

**Published:** 2018-02-20

**Authors:** Tessa van Dijk, Jan-Dirk Vermeij, Silvana van Koningsbruggen, Phillis Lakeman, Frank Baas, Bwee Tien Poll-The

**Affiliations:** 1 Department of Clinical Genetics, Leiden University Medical Center, Leiden, The Netherlands; 2 Department of Clinical Genetics, Academic Medical Center, University of Amsterdam, Amsterdam, The Netherlands; 3 Department of Neurology, Academic Medical Center, University of Amsterdam, 1105 AZ Amsterdam, The Netherlands; 4 Department of Pediatric Neurology, Academic Medical Center, University of Amsterdam, 1105 AZ Amsterdam, The Netherlands

## Abstract

Mutations in the *SEPSECS* gene are associated with pontocerebellar hypoplasia type 2D. Pontocerebellar hypoplasia (PCH) is a heterogeneous group of rare autosomal recessive neurodegenerative disorders, mainly affecting pons and cerebellum. Patients have severe motor and cognitive impairments and often die during infancy. Here, we report a 23-year-old woman with slowly progressive cerebellar ataxia and cognitive impairment, in whom a homozygous missense mutation in the *SEPSECS* gene (c.1321G>A; p.Gly441Arg) was identified with whole exome sequencing. Our findings underline that defects in selenoprotein synthesis can also result in milder cerebellar atrophy presenting at a later age.

**Electronic supplementary material:**

The online version of this article (10.1007/s10545-018-0151-x) contains supplementary material, which is available to authorized users.

A 23-year-old woman presented with a history of intellectual disability, ataxia and progressive decline of motor function after initial normal development. Neurological examination revealed microcephaly (head circumference is 50.5 cm; −3.6SD), horizontal nystagmus, evident dysmetria and a broad based gait with postural instability. Magnetic resonance imaging (MRI) of the brain at the age of 16 years showed mild cerebellar atrophy (not shown), which had progressed at the age of 23 (Fig. 1b and Fig. 1a shows control image for comparison). Several metabolic (e.g. congenital disorders of glycosylation type 1a) and genetic (e.g. Friedreich ataxia) disorders were excluded (see Supplementary material for extended case description).

**Fig. 1:**
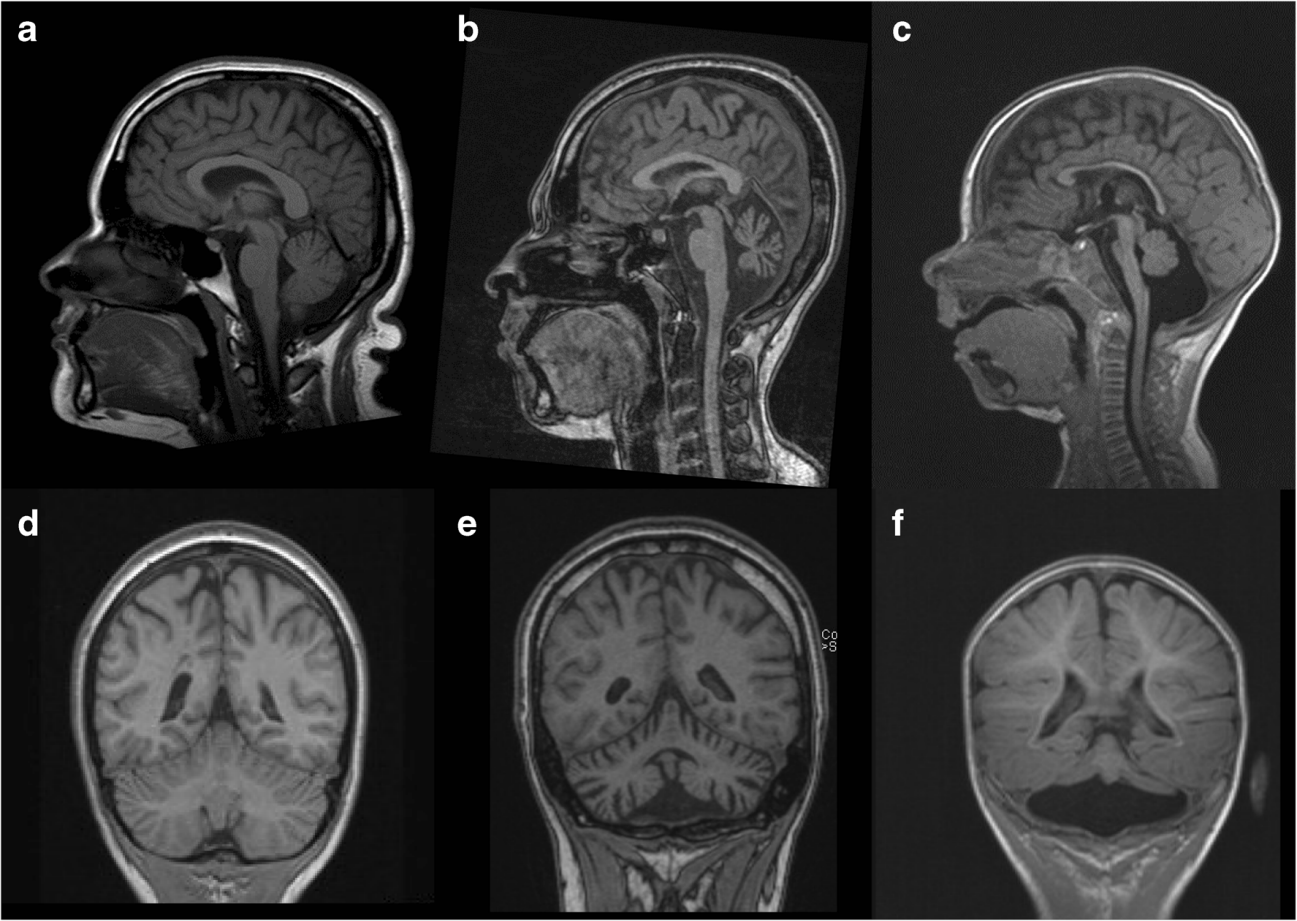
Brain MRI showing cerebellar atrophy in our patient, compared to a control and a PCH2A patient. (**a**) T1-weighted midsagittal image of a control. (**b**) T1-weighted midsagittal image of our patient, aged 23. Enlarged extracerebellar liquor spaces indicate atrophy of cerebellar vermis. The pons is normal. (**c**) Midsaggital MPR-image of a PCH2A patient at 6 months of age shows severe flattening of the ventral pons and hypoplasia of the cerebellar vermis. (**d**) MPR coronal image of control. (**e**) MPR coronal image or our patient shows atrophy of cerebellar hemispheres. (**f**) MPR coronal image of PCH2A patient indicates severe cerebellar hypoplasia with relative sparing of the vermis, resulting in a typical dragonfly pattern

Whole exome sequencing (trio analysis) revealed a homozygous missense variant: c. 1321G > A; p.(Gly441Arg) in exon 11 of the O-Phosphoseryl-tRNA selenocysteine tRNA synthase (*SEPSECS, NM_016955.3*) gene in the patient. Both parents were heterozygous carriers of this variant, that was predicted pathogenic by various in silico prediction programs (see Supplementary material).

*SEPSECS* mutations are associated with pontocerebellar hypoplasia type 2D (PCH2D). PCH2 is a prenatal onset neurodegenerative disorder characterized by severe hypoplasia of cerebellum and pons (Fig. 1c). PCH2D is a very rare subtype of PCH2 characterized by profound intellectual disability, progressive microcephaly, spasticity, epilepsy and progressive cerebral and cerebellar atrophy (Agamy & Zeev 2010). Additional features like axonal neuropathy, optic nerve atrophy, secondary mitochondrial dysfunction and early onset epileptic encephalopathy with burst suppression were later reported in patients with *SEPSECS* mutations (Anttonen et al 2015; Pavlidou et al 2016; Olson et al 2017). Disease onset was early and psychomotor development was severely delayed or absent in these patients. Our findings indicate that *SEPSECS* mutations can also give rise to milder and later onset neurodegeneration. It is currently unknown why our patient’s phenotype is so much milder. Possibly, the Gly441Arg missense mutation, which is located in the last exon of *SEPSECS*, results in a SEPSECS protein with a higher residual activity. This milder clinical presentation of *SEPSECS* mutations should be kept in mind in clinical care and when interpreting *SEPSECS* variants identified by whole exome sequencing.

## Electronic supplementary material

ESM 1 (DOC 26 kb)
