# Supplementary material for: A SEPSECS mutation in a 23-year-old woman with microcephaly and progressive cerebellar ataxia
Source: J Inherit Metab Dis. 2018 Feb 20;41(5):897–8. doi: 10.1007/s10545-018-0151-x (PMC6133186; doi:10.1007/s10545-018-0151-x)
Supplement: Supplementary file 1 — (DOC 26 kb) [file 10545_2018_151_MOESM1_ESM.doc]

**Supplementary Material**

**Case report**

A 23-year-old woman presented with a history of intellectual disability, ataxia and progressive decline of motor function after initial normal development. She started walking at 12 months of age and at the age of 2 years she could speak some single words. At the age of 3, speech did not develop any further and cognitive impairment was noted. By the age of 14, a progressive decline in motor function became apparent: she lost the ability to jump and run, and is currently only able to walk short distances with frequent stumbling. She communicates with pictograms and gestures. Her parents are consanguineous. Current head circumference is 50.5 cm (-3.6SD). She has spontaneous convergence of the eyes with a third degree horizontal nystagmus. Strength and sensation are normal, with decreased deep tendon reflexes and normal plantar reflexes. Point-to-point movement evaluation and heel to shin coordination show evident symmetric dysmetria. Gait is broad based with postural instability. Tandem walking is impossible. Electromyography (EMG) shows no indication of polyneuropathy. Metabolic screening of blood and urine, including amino acids, acylcarnitines and very long chain fatty acids is normal. Isoelectric focusing of transferrines is normal as well. Magnetic Resonance Imaging (MRI) of the brain at the age of 16 showed mild cerebellar atrophy (not shown). A second brain MRI at the age of 23 years showed progression of cerebellar atrophy (Fig. 1B and Fig. 1A shows control image for comparison) but was still very mild compared to the severe cerebellar hypoplasia seen in PCH2A (Fig. 1C). Pontine structures were normal. Biochemical and genetic testing for several types of spinocerebellar ataxia, Friedreich ataxia and congenital disorder of glycosylation type 1a (CDG1A) were all negative. Array CGH analysis identified no significant chromosomal microdeletions- or duplications.

**Whole exome sequencing (WES) and genetic analysis**

*WES*

DNA was extracted from peripheral blood samples using standard methods. Whole Exome Sequencing (WES) was performed on DNA of the patient and both parents. Library preparation and capture were carried out using the Kapa HTP kit (Illumina, San Diego, CA, USA) and the SeqCap EZ MedExome (Roche NimbleGen Madison, WI, USA), respectively. Sequencing of resulting libraries was performed on an Illumina HiSeq2500 HTv4 (Illumina, San Diego, CA, USA) with paired-end 125-bp reads. Reads were aligned to the human reference genome (h19) with BWAMEM (bio-bwa.sourceforge.net/). Variant calling was done using the GATK3.2 software package (www.broadinstitute.org/gatk/ ) and filtering was done with Cartagenia Bench Lab NGS (Agilent). Exclusion criteria were < 5 reads or a frequency higher than 1% in public (ESP, dbSNP, 1KG) and/or in house databases. *De novo*, homozygous or compound heterozygous variants that were located in exons or within +/- 6 nt in the intron were analyzed.

*Genetic analysis*

Trio analysis of the patient and her parents unexpectedly revealed a homozygous missense variant: c. 1321G>A in the O-Phosphoseryl-tRNA selenocysteine tRNA synthase gene (*SEPSECS, NM_016955.3*). Both parents were heterozygous carriers of this variant. Pathogenic variants in *SEPSECS* are associated with pontocerebellar hypoplasia type 2D (PCH2D). This variant results in an amino acid substitution of a highly conserved glycine by arginine (p.(Gly441Arg)) in exon 11. The variant was predicted pathogenic by various in silico prediction programs (i.e. SIFT, Polyphen) and is very rare (only 2 heterozygous calls in 121164 alleles in the Exac database (http://exac.broadinstitute.org)).
